# Enhanced Transmit Efficiency of a Loop Array Combined With Out‐of‐Phase Passive Dipoles for Thoracic Spinal Cord MRI at 7T

**DOI:** 10.1002/mrm.70517

**Published:** 2026-07-26

**Authors:** Hugo Amat, Marc Dubois, Aurelien Destruel, Sajad Hosseinnezhadian, Djamel Berrahou, Charles Betemps, Amira Trabelsi, David Bendahan, Stefan Enoch, Virginie Callot, Redha Abdeddaim

**Affiliations:** ^1^ Multiwave Technologies Marseille France; ^2^ Aix Marseille Univ, CNRS, Centrale Med, Institut Fresnel Marseille France; ^3^ Aix Marseille Univ, CNRS, CRMBM Marseille France; ^4^ AP‐HM, Hôpital de la Timone, CEMEREM Marseille France

**Keywords:** 7T, passive dipoles, passive shimming, radiofrequency coil, spinal cord, transmit array

## Abstract

**Purpose:**

The design of radiofrequency (RF) coils tailored for the target anatomy is necessary to overcome the transmit field homogeneity and penetration issues at Ultra‐High‐Field (UHF). For the spinal cord, the optimal design has not yet been determined. This work intends to demonstrate the added value of a pair of passive dipoles placed under a conventional thoracic spine 4 × 2‐loop array.

**Methods:**

The conventional loop array produces a broad transmit field with unnecessary lateral spread. To address this, two passive dipoles were incorporated and tuned out‐of‐phase to actively suppress these lateral side‐lobes and increase field penetration the spinal cord region. The design was numerically optimised using a homogeneous phantom, followed by human voxel model simulations to evaluate performance and specific absorption rate (SAR). Finally, a prototype was tested in a 7T scanner using a homogeneous phantom to acquire experimental $B_{1}^{+}$ maps.

**Results:**

Human voxel model simulations demonstrated that the passive dipoles improve the $B_{1}^{+}$ transmit field distribution and predicted a 35% gain in SAR efficiency in the spinal cord region. Correspondingly, phantom experiments showed a 25% gain in transmit efficiency and 15% gain in homogeneity in a selected region of interest compared to the loop‐array‐only reference.

**Conclusion:**

Incorporating passive dipoles effectively suppresses unwanted lateral field spread, increasing penetration depth and $B_{1}^{+}$ efficiency in the thoracic spinal cord region. While homogeneous phantom results offer proof of concept, numerical simulations suggest reliable performance in realistic spine anatomy as well as substantial SAR reduction.

## 1 Introduction

Magnetic resonance imaging (MRI) is a modality of choice for spinal cord imaging as it offers good visualisation of soft tissues. As signal‐to‐noise ratio (SNR) scales with the main $B_{0}$ field strength [1], ultra‐high‐field (UHF) 7T MRI has been expected to offer improved spatial resolution and new unique contrasts as compared to conventional field (1.5T and 3T) MRI. A better visualisation of small lesions and structural changes together with improved functional quantitative MRI have been advocated [2, 3, 4]. However, UHF MRI is related to a wavelength shortening thereby leading to **B**_**1**_ inhomogeneities [5]. Specific absorption rate (SAR) is another issue given that power deposition also scales with $B_{0}$ [6, 7]. Overall, whole‐body coils conventionally used at 1.5T and 3T are no longer used at 7T and local coil arrays adapted to the target anatomy should be designed [8].

This work focuses on the imaging of the thoracic spine which has been scarcely assessed so far as compared to cervical levels [3]. UHF MRI of this region is clinically relevant [9] to detect small lesions or tissue impairments that would be invisible at lower field strengths, better quantify disease burden and improve diagnosis, prognosis, and follow‐up in both degenerative and traumatic spinal cord pathologies [10, 11, 12].

From an anatomical perspective, thoracic spinal cord covers a 25 to 30 cm section, lies 4 to 6 cm deep under the skin [13] with a 6 mm average diameter (12 mm for the dural sac) [14]. Consequently, RF hardware designed for this region must satisfy specific requirements: provide deep penetration to reach the cord, maintain an extended longitudinal field of view (FOV) to cover the relevant region, and operate robustly in a torso environment prone to high **B**_**1**_ inhomogeneities. To meet these demands, RF coils proposed for the thoracic spinal cord region [15, 16, 17, 18, 19, 20, 21, 22] follow its elongated geometry. Transceive loop arrays organised in one row [16] or two rows [15, 17, 19] have been reported. Tx quadrature coil with one row of Rx loop array has also been designed [18]. Recent designs included dual Tx dipoles [20, 21] and microstrip transmission line arrays [22]. However, no consensus has been reached so far regarding the optimal design of the RF system (transmit and/or receive coils) for the spinal cord region [23].

Increasing the number of Rx elements allows for the use of parallel imaging techniques that can improve the SNR [24, 25]. However, this is mostly true for shallow and peripheral regions given that for deeper targets like the thoracic spine, Rx loops need careful positioning and sufficient diameter [16]. Concurrently multiple Tx elements enable **B**_**1**_ shimming as well as parallel transmission (pTx) [26] which are of great interest if one intends to reduce $B_{1}^{+}$ inhomogeneities in long regions such as the thoracic spine [27] and can also lower SAR [28, 29, 30]. In both cases optimal decoupling between the elements is necessary, to reduce noise correlation [25] or increase degrees of freedom [31].

Focusing on Tx designs, it can be noted that dipole elements have been recognised as well‐suited for deeper targets and torso imaging at UHF [32, 33] as well as for cervical spine [34]. One interesting idea is the combination of loop and dipole currents, either with superposition of the two elements [35], even‐mode dual dipoles [36] or unbalanced loops [37]. These solutions can optimise performance for both shallow and deeper levels, increase channel density, decrease SAR and offer a better stability. Another recent development is the use of passively fed coupled dipoles [38, 39] that can improve SAR performance by containment of the electric fields. More generally, in UHF MRI, the concept of using passive elements to tailor the transmit field was mainly explored with volumetric head coils, where high permittivity materials [40] or metasurfaces [41, 42, 43] are added to correct the **B**_**1**_ pattern. However, it has been shown that dielectric resonators [44], dipole arrays [45] or split‐ring metasurfaces [46] can also be used in combination with surface loop coils to improve their performance.

While these studies demonstrate the potential of passive structures to reshape the transmit field, they primarily utilise bulk dielectrics or metasurfaces and focus on single transmit loops. To the authors' knowledge, the specific integration of a multi‐element loop array with passive dipole elements has not been previously explored. Furthermore, while conventional hybrid loop‐dipole arrays can achieve global field shaping, they rely entirely on active elements requiring complex feeding networks. To address these gaps—and the hardware constraints of standard 7T systems—a novel design tailored for thoracic spinal cord MRI is proposed here. While it has been shown that dipole antennas offer superior transmit performance for targets deeper than 6 cm [47], their geometry limits the achievable element density along the vertebral column. For an elongated posterior target like the thoracic spine, an active dipole array is practically limited to two transmit channels, severely restricting the degrees of freedom available for subject‐specific shimming and parallel transmit (pTx). For this reason, an 8‐loop array configuration is chosen to maximise element density and take advantage of robust geometric decoupling. Because many 7T systems are limited to 8 transmit channels, fully driving this loop array leaves no available channels for additional active elements. Consequently, passive dipoles are introduced here explicitly for macroscopic field shaping. Since a loop array inherently generates undesired $B_{1}^{+}$ lateral field spread outside of the thoracic spine region even with optimal shimming, combining the surface loops with these passive dipoles can correct this spread, concentrating the transmit field in the target area while simultaneously achieving substantial SAR reduction.

The presented coil was designed with an optimised fixed RF shim. To understand how to optimally position the passive structures, the baseline loop array was first analyzed using a simplified theoretical model. Specifically, the optimised RF shim creates current patterns in the loops that resemble those produced by a set of theoretical active dipoles. By simulating this theoretical active dipole model, it was possible to clearly identify which specific current elements were responsible for the unwanted lateral magnetic field spread. Based on this theoretical insight, appropriately tuned passive dipoles were added to the loop array to compensate for those undesirable current contributions, responding out‐of‐phase to their associated magnetic fields. This study demonstrates that this strategy successfully reshapes the field, allowing for better $B_{1}^{+}$ homogeneity, higher transmit efficiency and SAR reduction compared to a loop array alone.

## 2 Methods

### 2.1 Design of the Array and Passive Dipoles

The loop array used in this study is based on the design of Kraff et al. [15], consisting of two shifted rows of four loops each, oriented in the longitudinal direction. Each loop has a 10 cm diameter in accordance with optimal dimensions found in literature [16]. In other words, it is large enough to cover the target anatomy and reach sufficient penetration depth. A larger size would increase the field inhomogeneities [48], would have a higher coupling, and would make the pTx control coarser. The loop tracks have a width of 2 mm and a thickness of 35 μm and were fabricated by computer numerical control milling of a copper‐clad FR4 plate. Each loop was divided into four segments by three 2.7 pF capacitors to reduce the electrical length and avoid field asymmetries due to phase variations [48, 49]. At the feeding gap, individual loops were interfaced with a 50 Ω non‐magnetic coaxial cable (HY223, Hytem, France) via a balanced L‐section matching network [50]. This circuit comprised a variable tuning capacitor connected in parallel to the port (1.5–50 pF, operating predominantly around 1.5–3 pF) and two fixed matching capacitors connected in series. To electrically balance the circuit, the matching capacitance was split evenly between these two series components, with values ranging from 20 to 33 pF depending on the specific electromagnetic environment of each loop (yielding an equivalent series matching capacitance of 10 to 16.5 pF). The loops were tuned and matched at 297.2 MHz in the presence of a homogeneous phantom (350 × 175 × 480 mm^3^, εr = 58.2, *σ* = 0.92 S·m^−1^). Floating cable traps [51] were added on the cables to suppress common mode currents. The Q factor of the loaded/unloaded loop was evaluated with a dual pick‐up probe [52].

**FIGURE 1:**
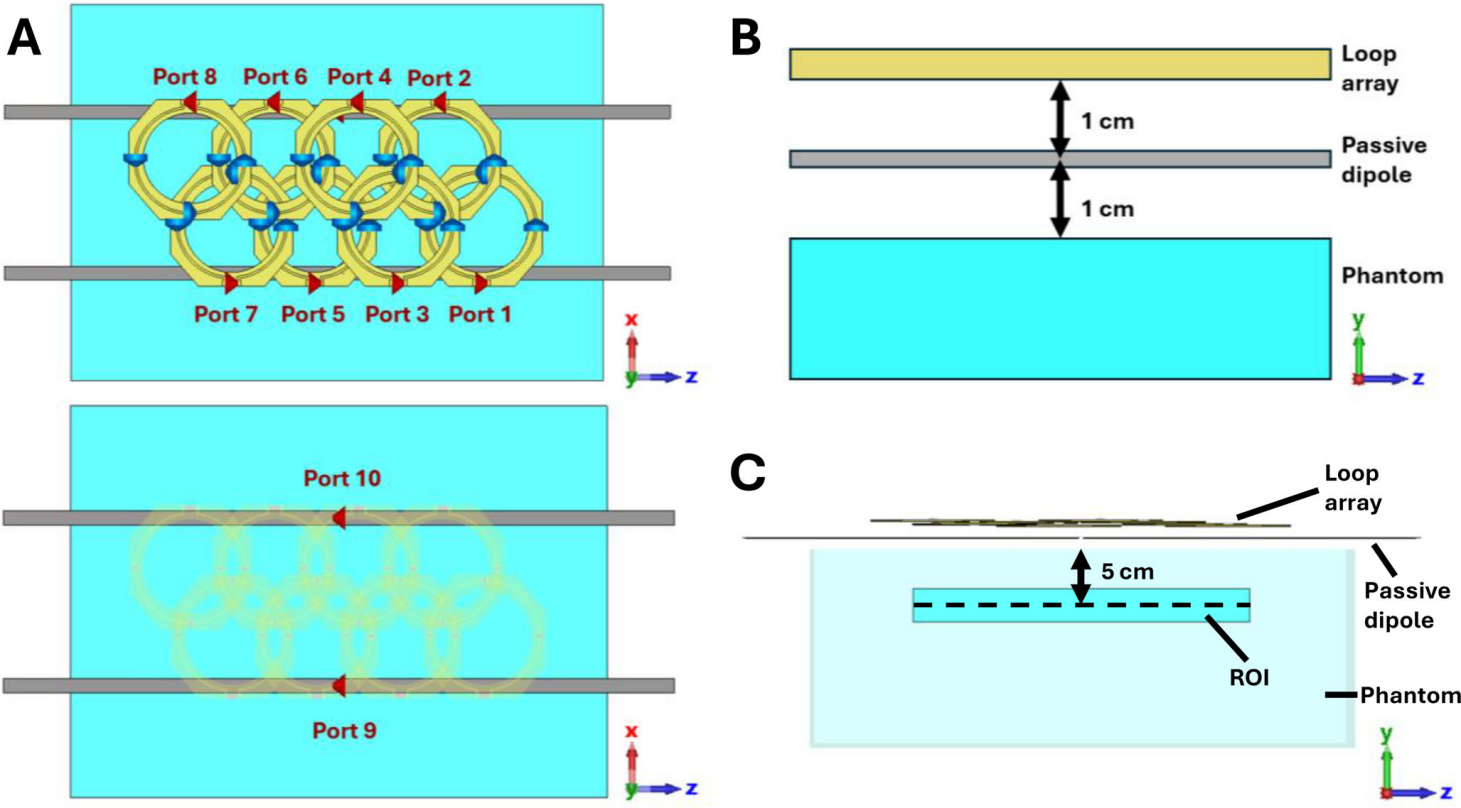
(A) Schematic view of the setup, with the different ports, showing capacitors (blue) and feeding ports (red). The two passive dipoles were not included in the array‐only reference case. (B) Position of the two passive dipoles between the array and the phantom. (C) Position of the ROI mimicking the thoracic spinal cord.

The eight loops were assembled in the desired array configuration (Figure 1A) on a foam support over the phantom while monitoring the S‐parameters with a Vector Network Analyzer (Keysight, E5063A). Decoupling was achieved by partial overlapping, and the tuning and matching were adjusted to adapt to the assembled array.

Pairs of passive dipoles consisting of copper strips (12.5 mm width, 35 μm thickness) of different lengths (500/600/700 mm) were fabricated on 0.8‐mm‐thick FR4 supports. The spacing between dipoles was fixed at 150 mm (matching the loop array width), and dipoles were segmented to allow the addition of a 10‐pF capacitance.

### 2.2 Numerical Simulations

Numerical simulations were performed with CST Studio Suite 2024, using the time‐domain solver (Finite Integration Technique). The 8‐loop array was modelised in 3D with its conductive elements and substrate, and the segmentation capacitors were added as lumped elements. This constituted the array‐only reference case. For the cases featuring passive dipoles, two dipoles of tunable length were inserted 1 cm under the outer edges of the array as shown in Figure 1. The *X*, *Y* and *Z* axes correspond respectively to the left–right, posterior–anterior and inferior–superior axes. Note that for the homogeneous phantom simulations, the *Y*‐axis origin is defined at the phantom surface, whereas voxel model simulations and experimental evaluations adopt standard clinical scanner coordinates (*Y*‐axis centred within the subject).

Ports 1 to 8 were inserted at the feeding point of the loops. For simulations including the two passive dipoles (Figure 1A,B), ports 9 and 10 were defined at the segmentation gap of each dipole. Although the dipoles are meant to be passive elements, those last two ports were introduced solely to facilitate tuning and current monitoring via co‐simulation. The tuning and matching networks for each loop port were then modeled within the circuit co‐simulation environment. They consisted of all‐capacitive L‐section networks using ideal lumped elements. The simulations were run with a −60 dB energy decay stopping criterion and the meshing was refined until results converged: the end mesh counted around 50 million cells.

Simulation studies served a twofold objective: elucidate the physical mechanisms concerning the behaviour of our structure and provide a high‐fidelity predictive model capable of estimation of the field metrics. They were divided into three parts:

The first part was dedicated to the choice and analysis of the static array excitation. For this, a homogeneous phantom, identical in size and properties to the one described in the design section, was placed 2 cm under the loop array. A 30 × 30 × 300 mm^3^ region of interest (ROI) was defined around what would be the hypothetical position of the thoracic spinal cord, under the centre of the array and 5 cm deep into the phantom (Figure 1C). A set of excitations with varying phase shifts between the two rows of loops was simulated to verify that the excitation proposed by Kraff et al. was the best fit for the present case. This specific excitation scheme was then selected for the remainder of the study: it consisted of a constant amplitude for all ports and a 180° phase shift between the two loop rows, creating currents flowing in the same direction in the central part of the array where the two rows overlap. To better understand the current patterns created by this excitation, a simplified 4‐dipole model of the array was subsequently simulated. This model consisted of 4 four active dipoles that mimic the current distributions in the loops. These active dipoles were driven with ideal current sources and reproduced the relative distribution of current magnitudes extracted from the loop array simulation. The $B_{1}^{+}$ maps from the loop array and from the 4‐dipole model simulations were extracted and compared side by side to demonstrate the similarities between both configurations. As expected, both configurations exhibited an overly broad transmit field. Passive dipoles, identical to those shown in Figure 1, were then added to both loop array and 4‐dipole model. The resulting $B_{1}^{+}$ maps were then compared to the baseline (dipole‐less) cases to evaluate the field‐shaping effect of these passive structures in suppressing the undesired lateral spread and improving penetration in the ROI.

In the second part of the numerical simulations, the loop array, homogeneous phantom and passive dipoles were kept as displayed in Figure 1. A parametric study was conducted to evaluate the influence of passive dipole length. Its first objective was to study the transfer of currents between the loops and the dipoles. Accordingly, currents were monitored in port 4 (feeding point of a loop) and port 10 (nearest dipole). The second task was to evaluate the $B_{1}^{+}$ field inside the ROI defined above while varying the passive dipole length by monitoring the average $B_{1}^{+}$ efficiency ($B_{1}^{+}$ over the square root of stimulated power) and the $B_{1}^{+}$ coefficient of variation (CoV, defined as the $B_{1}^{+}$ standard deviation over the $B_{1}^{+}$ average). It should be noted that the $B_{1}^{+}$ efficiency was normalised to the stimulated power and that the loop array was tuned and matched in presence of the passive dipoles.

The third part of the simulations was dedicated to the evaluation of the design on a voxel‐based representative human model (Gustav from the CST Voxel Family). A new voxel‐model specific ROI encompassing the thoracic spinal cord and its surroundings was defined (which can be seen in Figure 5). $B_{1}^{+}$ profiles obtained in the presence of the array with the best performing passive dipoles were compared to the reference case with the array only. To ensure a fair comparison, both configurations were shifted along the left–right axis to align the spine with the region of strongest $B_{1}^{+}$ generated by the coil. The maximum local SAR (SAR_10g_, averaged over 10 g, Reference IEC 60601‐2‐33) and the ‘SAR efficiency’ (defined as the ratio of the average $B_{1}^{+}$ in the voxel‐model ROI to the square root of maximum local SAR) were computed. SAR_10g_ was chosen as a metric instead of global SAR as it constitutes the limiting factor in body MRI with surface coils [53].

### 2.3 MR Experiments

The setup was experimentally tested for three different dipole sizes (500, 600 and 700 mm) and a reference case (without dipoles) in a 7T scanner (Magnetom TERRA, Siemens Healthcare, Erlangen, Germany) using a rectangular phantom filled with body mimicking solution (Body Liquid 300, MVG) of the same dimensions and properties as described above. An eight‐channel T/R switch box (Stark Contrast, Erlangen, Germany) was used as an interface connection between the array and the scanner.

Reflection and coupling data were acquired from the scanner monitoring system to serve as a qualitative indicator of array performance. The system reports a complex 8 × 8 matrix calculated from forward and reflected/coupled wave at the directional couplers. Although the measure isn't calibrated to the coil elements (and thus those coefficients differ from standard S‐parameters), they effectively capture the relative variation in coupling magnitude between different volunteer setups. This allows for the identification of huge impedance mismatches or unsafe high‐coupling scenarios. Flip angle maps were acquired using a presaturated TurboFLASH (satTFL) sequence [54], employing a highly selective Shinnar‐Le Roux (SLR) saturation pulse prior to the TurboFLASH readout to specifically mitigate 2D slice cross‐interference [55] (FOV: 280 × 280 mm^2^, Matrix size: 56 × 56, Pixel size: 5 × 5 mm^2^, Orientation: axial, Number of slices: 64, Slice thickness: 2.5 mm, Spacing between slices: 5 mm, TR/TE: 17800/1.81 ms, Saturation FA: 60°, Excitation FA: 5°). These data were used to compute $B_{1}^{+}$ efficiency maps by scaling the known reference $B_{1}^{+}$ ($B_{1,\mathrm{ref}}^{+}$) and voltage (*V*_ref_) by the ratio between the measured (*α*_meas_) and the nominal (*α*_nom_) flip angles (Equation 1). Here *Z*_0_ is the reference impedance of 50 Ω, and *P* denotes the total forward transmit power delivered by the scanner to the transmit chain for the nominal flip angle.

(1) $$\frac{B_{1}^{+}}{\sqrt{P}}=\frac{B_{1,\mathrm{ref}}^{+}}{\sqrt{V_{\mathrm{ref}}^{2}/Z_{0}}}\times \frac{\alpha_{\text{meas}}}{\alpha_{\mathrm{nom}}}$$

One‐dimensional $B_{1}^{+}$ profiles (averaged over slices within the ROI) along selected axes were extracted from experimental measurements and compared to CST simulation results. For this comparison, simulated $B_{1}^{+}$ fields were scaled by a factor of 0.8 to account for the losses in the RF transmission chain. This 0.8 factor results from the combination of a 0.875 gain factor (provided in the scanner documentation) corresponding to the losses that occur between the amplifiers output and the scanner plug, and an additional 0.91 gain factor (measured on the RF bench) between the scanner plug and the BNC ports of the T/R switch interface box.

Finally, to ensure the integration of passive dipoles did not degrade the array's receive capabilities, complementary experimental evaluations of parallel imaging stability (g‐factors), noise covariance and signal‐to‐noise ratio (SNR) were performed (detailed in the Supporting Information).

## 3 Results

### 3.1 Numerical Simulations

**FIGURE 2:**
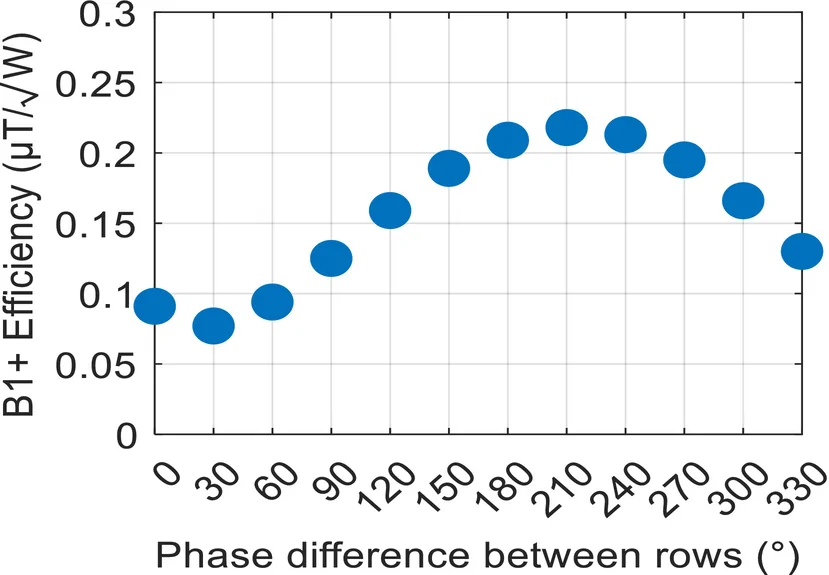
Average $B_{1}^{+}$ efficiency over the ROI for different phase shifts between the two loop rows.

For the reference case (without passive dipoles), different loop excitations were tested by varying the phase shift between the two rows while maintaining uniform amplitude. Figure 2 shows the $B_{1}^{+}$ efficiency averaged over the ROI for each phase difference. The $B_{1}^{+}$ efficiency was higher in the ROI when ports in different rows were in phase opposition. The maximum efficiency was reached for 210° phase difference, but a 180° phase shift (96% of maximum efficiency) was used as our default excitation to simplify the RF shim implementation.

**FIGURE 3:**
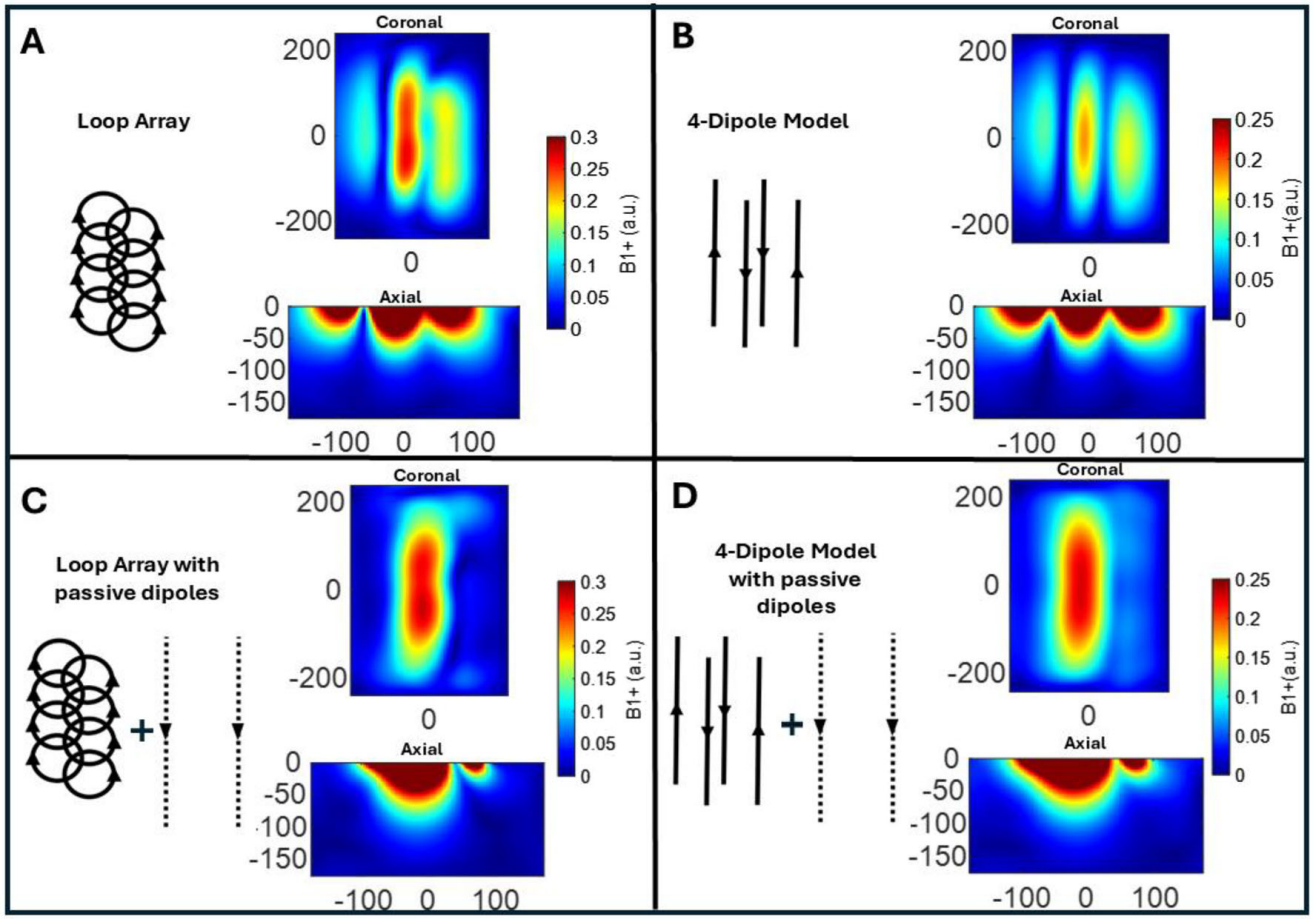
$B_{1}^{+}$ maps simulated in the homogeneous phantom in 4 different cases. (A) Loop array; (B) 4‐active‐dipole model; (C) Loop array with 600 mm passive dipoles; (D) 4‐active‐dipole **model** with 600 mm passive dipoles. In (B, D), the 4 dipoles were excited at their centre with ideal current sources of magnitude and phase comparable to those of the elements they represent in the loop array case. The scales for (A, C) and (B, D) are independent. Axis unit is mm. The displayed coronal plane is positioned at a depth of 5 cm inside the phantom (or 7 cm under the array) while the axial plane stands under the middle of the array.

Figure 3A shows the $B_{1}^{+}$ obtained with this RF shim in a coronal plane at the depth of the ROI and a central axial plane. A central area of high magnitude is present along the longitudinal axis, as well as asymmetrical lateral lobes. The $B_{1}^{+}$ field generated by our 4‐dipole model is plotted in Figure 3B. Its distribution is similar to the one produced by the loop array, indicating that the simplified dipole model is able to replicate the behaviour of the array. In Figure 3C and Figure 3D, pairs of passive dipoles were added (to the loop array and the 4‐dipole model respectively) and the displayed $B_{1}^{+}$ profiles allow us to visualise how the passive dipoles focus the field in a central region along the longitudinal axis, extending the area of high magnitude and eliminating the lateral lobes.

**FIGURE 4:**
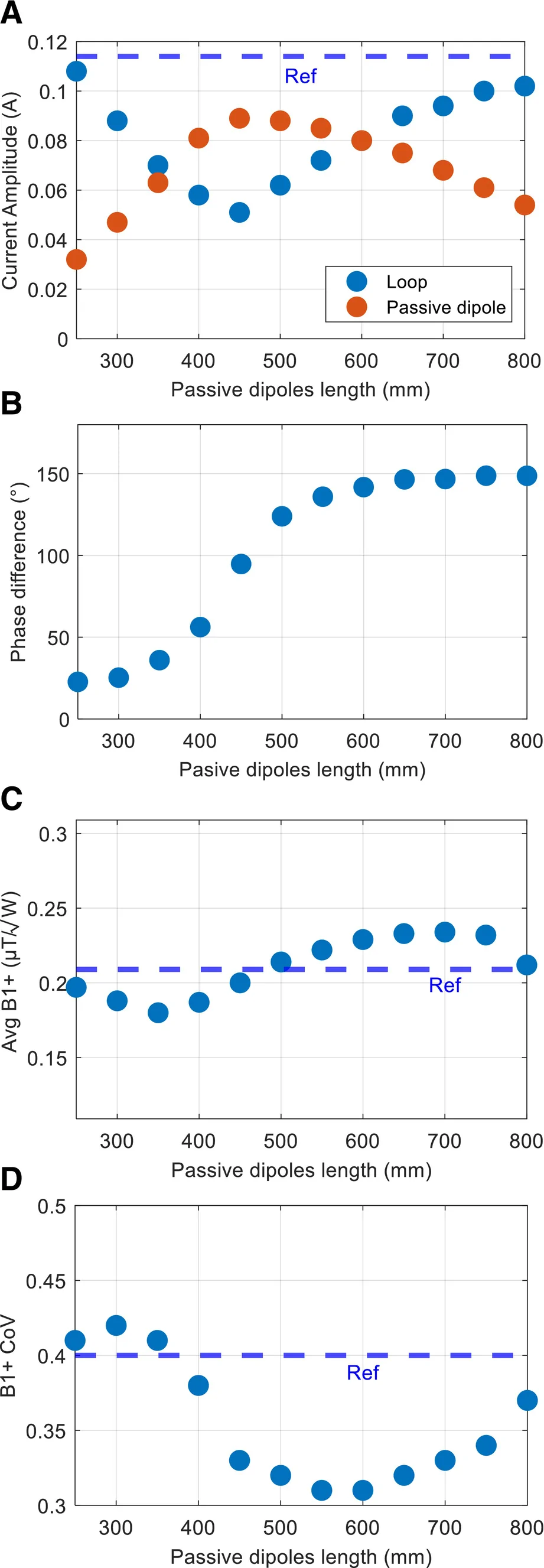
Evolution of current amplitude (A) and phase difference (B) between one loop feeding port (port 4) and the centre of the passive dipole placed under it (port 10), for different passive dipole lengths. Average $B_{1}^{+}$ efficiency (C) and $B_{1}^{+}$ CoV (D) in the ROI for different passive dipole lengths. The blue dashed lines mark the reference levels corresponding to the loop array alone case.

The parametric study on passive dipole length is shown in Figure 4, displaying the evolutions of currents amplitudes and phase differences for loops and passive dipoles. As the dipole length increases, current distribution shifts between the two elements (Figure 4A) and currents progressively evolve from in‐phase to out‐of‐phase (Figure 4B). The maximum current intensity in dipoles and the phase shift occur when the passive dipoles are close to their effective resonant length (approximately 450 mm in the fully coupled, loaded state).

The $B_{1}^{+}$ efficiency (Figure 4C) and homogeneity (Figure 4D) were also affected by dipole length. The segmentation of the passive dipoles in two halves allowed lumped elements to be inserted, which were used to control their resonant length (at which the phase shift occurs). Adding a 10‐pF capacitor helped maintain a longer physical dipole length, which improved homogeneity. The average $B_{1}^{+}$ efficiency in the ROI area was maximised (+12% compared to the reference level) for 700 mm dipoles and the CoV minimised (−23%) for 550 and 600 mm dipoles (Figure 4C,D). Those results demonstrate that the most favourable cases for both $B_{1}^{+}$ efficiency and CoV are those obtained with passive dipoles longer than ‘resonant length’, when the loop and passive dipole currents are out of phase. The 500, 600 and 700‐mm cases were selected for experimental investigations, being the most promising. Furthermore, a supplementary sensitivity analysis on the lateral spacing between the dipoles (Supporting Information S1) confirmed that the chosen 150 mm spacing—designed to match the active loop array's outer edges—provides an optimal trade‐off, yielding the highest SAR efficiency compared to narrower (130 mm) or wider (170 mm) spacings.

**FIGURE 5:**
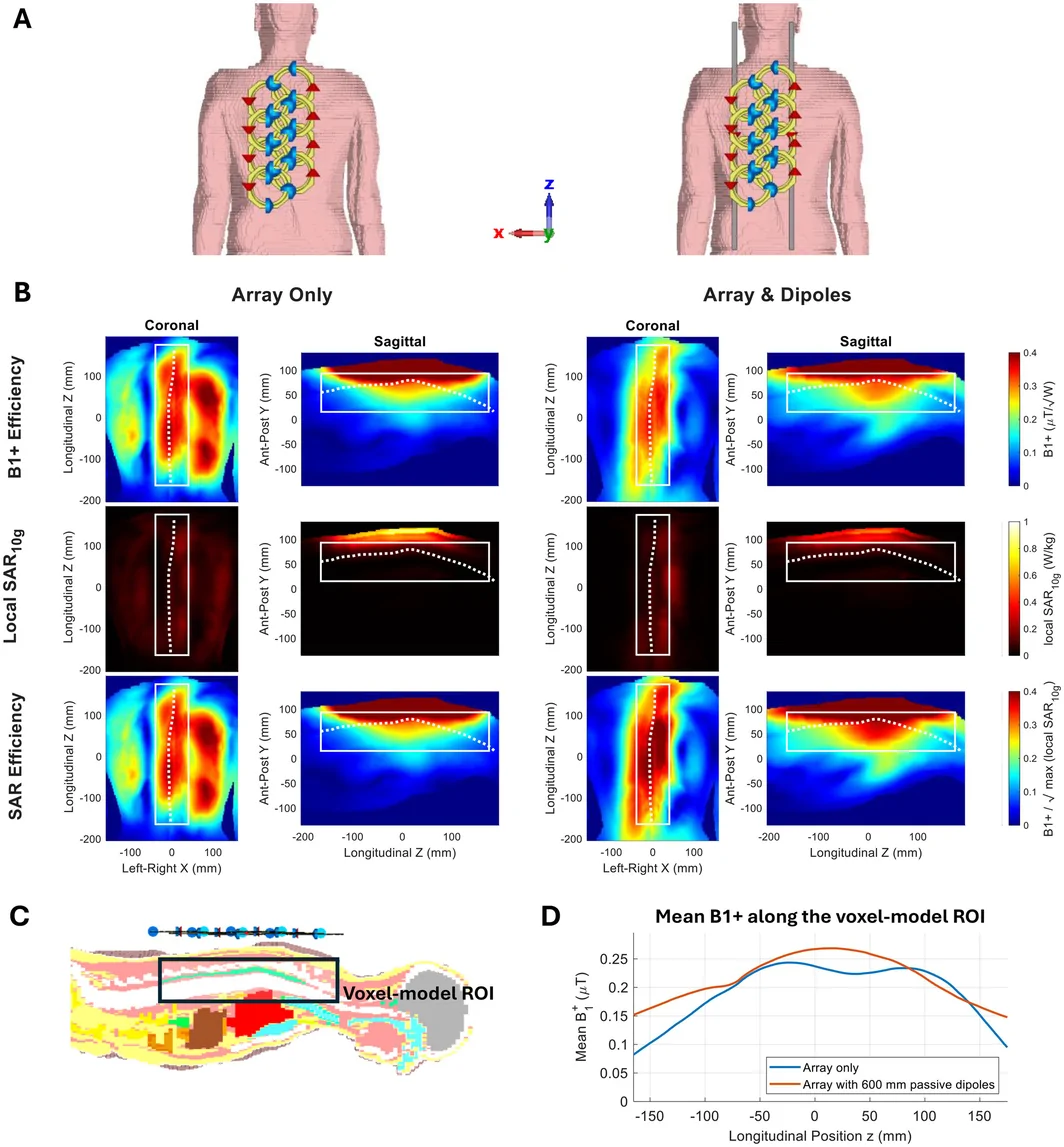
Voxel model simulation results. (A) 3D view of the simulated setup: The array is positioned on the posterior side of the Gustav voxel model, without (left) and with (right) the 600 mm passive dipoles. (B) $B_{1}^{+}$ efficiency, local SAR_10g_ and SAR efficiency maps in the voxel model for the array only (left) and the combined array with passive dipoles (right). The coronal plane is located at a depth of 7 cm anterior to the loop array, and the sagittal plane corresponds to the mid‐sagittal plane of the model. The white rectangle indicates the boundaries of the voxel‐model ROI, and the dotted white line represents the 2D projection of the spinal cord. (C) Mid‐sagittal cross‐section of the Gustav model. The superimposed box shows the position of the voxel‐model ROI encompassing the spine area. (D) Longitudinal profile of the mean field. At each longitudinal position (*z*), the mean was calculated as the spatial average of the field magnitude over the transverse plane (*x*, *y*) within the boundaries of the voxel‐model ROI. The axes origin is here located at the centre of the voxel model, differing from the surface‐referenced origin used in the homogeneous phantom figures.

For simulations with the Gustav human voxel model, 600 mm dipoles were selected. In order to align the central region of B_1_^+^ illumination with the spinal cord, the antenna was shifted from the middle (in the *X* or left–right axis) of the model by 15 mm for the array alone (Figure 5A) and 20 mm for the array with passive dipoles (Figure 5B). The corresponding $B_{1}^{+}$ maps are displayed for a coronal plane (Figure 5C,D) 7 cm under the array and a midsagittal plane (corresponding to the geometric centre and symmetry axis of the voxel model) (Figure 5E,F). As in the homogeneous phantom case, the addition of the dipoles eliminates the lateral contributions of the field and extends the illumination in the longitudinal direction.

As shown in Table 1, for the best‐performing 600‐mm case, the addition of passive dipoles improves the transmit metrics of the array. Note that the ‘stimulated power’ corresponds to the total input power while the ‘accepted power’ accounts for power dissipated in the simulation ports due to reflection and coupling. Despite a 24% reduction in accepted power, the passive dipoles yielded an 11% gain in B1+ efficiency, a 32% reduction in CoV, and a 35% gain in SAR efficiency.

**TABLE 1:** Comparison of $B_{1}^{+}$ and SAR metrics in the Gustav voxel model. $B_{1}^{+}$ efficiency, CoV and SAR efficiency are calculated as described in the ‘Methods’ section; the voxel‐model ROI used here is plotted in Figure 5.

	Loop array only	Loop array with 600 mm passive dipoles
Stimulated power (W)	1.0	1.0
Accepted power (W)	0.93	0.71
Average $B_{1}^{+}$ efficiency in voxel‐model ROI ($\mathrm{\mu T}/\sqrt{W}$)	0.194	0.216
CoV in voxel‐model ROI	0.56	0.38
Peak local SAR_10g_ for whole phantom (W/kg)	1.01	0.69
SAR efficiency ($\mathrm{\mu T}/\sqrt{W/\mathrm{kg}}$)	0.193	0.260

To further evaluate the robustness of this design to variations in patient positioning and padding thickness, a numerical sensitivity analysis of the airgap between the dipoles and the subject was performed (Supporting Information S2). The results demonstrate that the coil's $B_{1}^{+}$ efficiency and maximum local SAR remain highly stable and within safety limits across airgaps ranging from 2 to 15 mm.

### 3.2 MR Experiments

**FIGURE 6:**
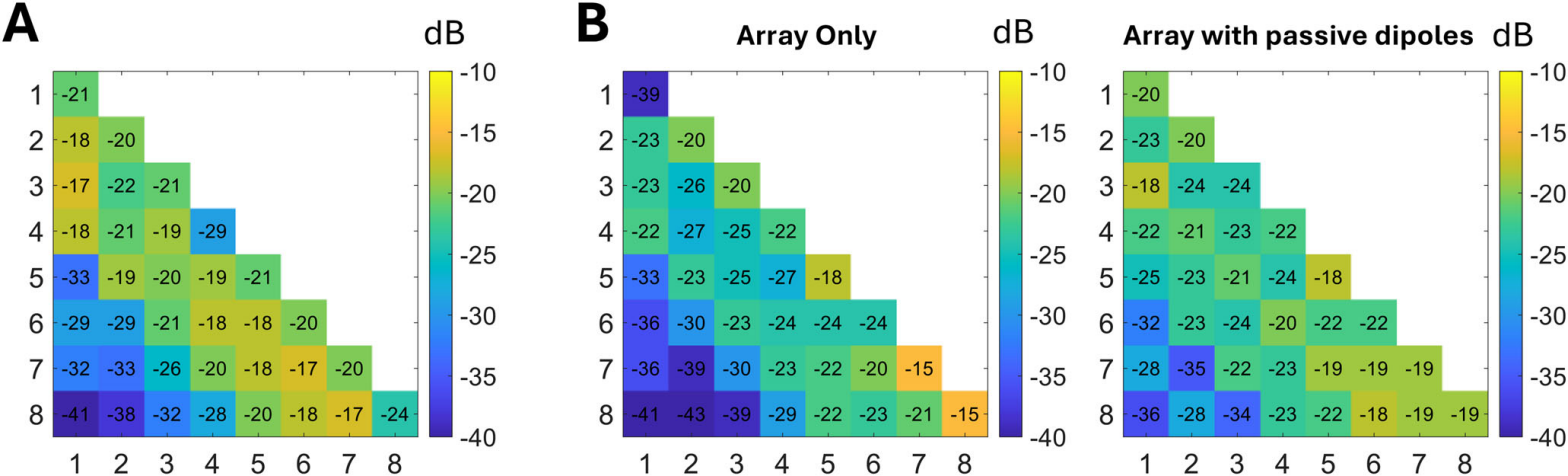
(A) S‐parameters acquired on the RF bench for the array without dipoles placed on the phantom. (B) Reflection and coupling parameters measured by the MRI scanner; the array was placed on the phantom without (left) and with (right) the 600 mm long passive dipoles.

The unloaded and loaded Q factors for a single loop were respectively 120 and 13 leading to an unloaded/loaded ratio of 9.2, indicating that the losses are sample dominated. S‐parameters acquired on the RF bench for the array without dipoles placed on the phantom are displayed in Figure 6A: the highest reflection is −15 dB and the highest coupling −18 dB. Reflection and coupling parameters acquired by the scanner are indicated in Figure 6B: it can be noted that the addition of the passive dipoles has a minor influence on those parameters, slightly increasing interelement coupling.

**FIGURE 7:**
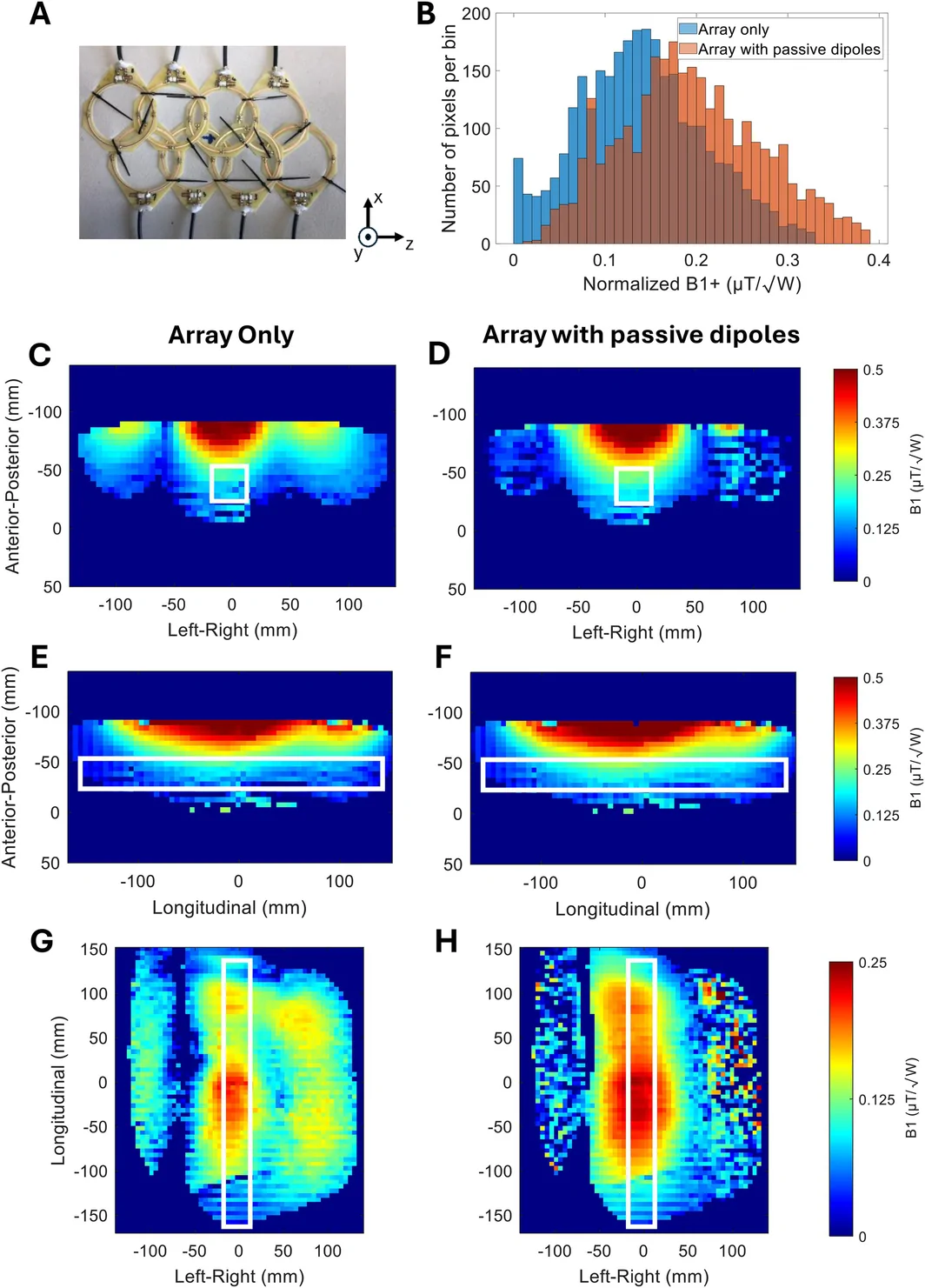
(A) View of the experimental array. The passive dipoles are placed under the foam support when used. (B) Histogram of the $B_{1}^{+}$ efficiency values for pixels inside the ROI, for the loop array only and for the loop array with 600 mm passive dipoles. (C–H) Experimental $B_{1}^{+}$ maps: (C, D) on a coronal plane 5 cm deep into the phantom, (E, F) on a central axial plane, (G, H) on a central sagittal plane. (C, E, G) for the loop array only; (D, F, H) for the loop array with 600 mm passive dipoles. The ROI is highlighted in white. The scanner's coordinate system is used here.

Figure 7 compares the results obtained with the experimental array alone (Figure 7A) and with the 600 mm passive dipoles. Normalised $B_{1}^{+}$ maps displayed in Figure 7C–H show that the addition of dipoles reshapes the transmit field as predicted by the simulations, while the histogram (Figure 7B) confirms an improved transmit efficiency in the ROI.

Table 2 shows a quantitative comparison between the different configurations, comparing the transmit efficiency and homogeneity in the ROI for the reference case and the three tested passive dipole sets. The 600 mm case performed better on both metrics: +25% average $B_{1}^{+}$ efficiency and −16% CoV compared to the reference case.

**TABLE 2:** Comparison of B_1_^+^ metrics in the ROI for the reference case and different passive dipole lengths, with the corresponding relative change (%) when comparing to the no‐dipole reference case.

	Array only	Array with 500 mm passive dipoles	Array with 600 mm passive dipoles	Array with 700 mm passive dipoles
Average $B_{1}^{+}$ efficiency in ROI ($\mathrm{\mu T}/\sqrt{W}$)	0.140	0.156 (+11%)	0.175 (+25%)	0.162 (+15%)
CoV in ROI	0.49	0.44 (−10%)	0.41 (−16%)	0.42 (−14%)

**FIGURE 8:**
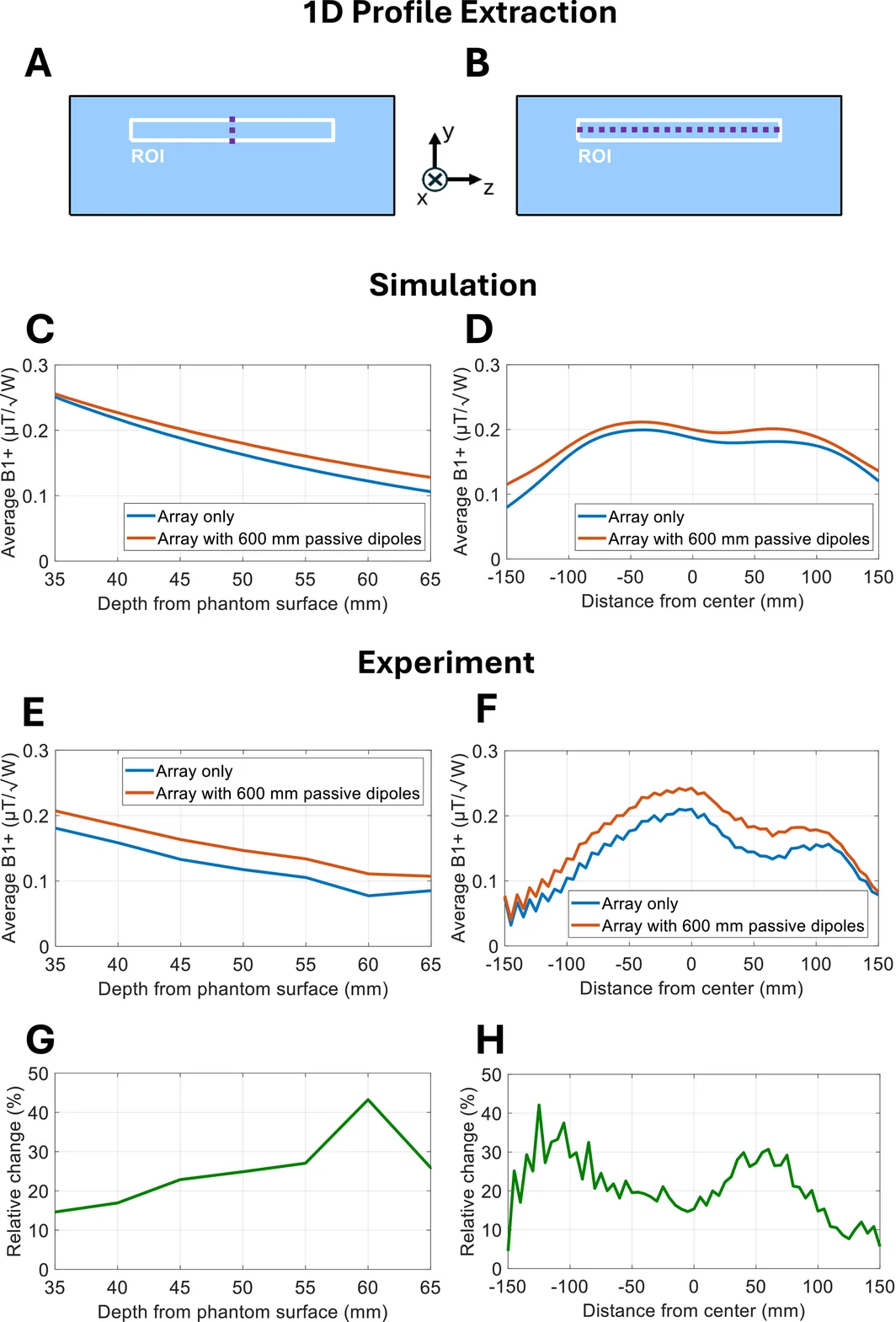
(A, B) Schematic sagittal representations of the phantom indicating the location of the ROI. The dotted curves indicate the profiles for data extraction along the antero‐posterior and longitudinal axes, respectively. (C, E) Mean $B_{1}^{+}$ profiles along the antero‐posterior direction (depth) for simulated and experimental data. At each depth position, the mean $B_{1}^{+}$ was calculated as the spatial average over the corresponding coronal plane (*x*, *z*) bounded by the ROI. The horizontal axis begins at 35 mm, matching the anterior boundary of the ROI to reflect the anatomical depth of the thoracic spinal cord. (D, F) Mean $B_{1}^{+}$ profiles along the longitudinal direction (*z*) for simulated and experimental data. At each longitudinal position, the mean $B_{1}^{+}$ was calculated as the spatial average over the corresponding transverse plane (*x*, *y*) bounded by the ROI. (G, H) Relative $B_{1}^{+}$ change between the loop array only and the loop array with 600 mm passive dipoles. Experimental results for antero‐posterior and longitudinal profiles, respectively.

Experimental $B_{1}^{+}$ profiles along antero‐posterior and longitudinal directions inside the ROI are compared with simulated ones on Figure 8 and indicate better penetration in the presence of the 600 mm passive dipoles.

Importantly, supplementary evaluations confirmed that the integration of passive dipoles successfully maintained the robust parallel imaging stability and intrinsic receive (Rx) sensitivity of the baseline loop array (see Supporting Information S3).

## 4 Discussion

The optimal excitation of the loop array (Figure 2), when the contribution of each row was in the same direction in the overlapping region, confirmed the conclusions of Kraff et al. [15]. Although this RF shim produces a central zone of intense and homogeneous $B_{1}^{+}$ along the longitudinal axis, which is desirable for spinal cord imaging, asymmetric ‘lobes’ are present on the left–right axis, with one more pronounced lobe on one side and a smaller lobe as well as an area of very weak $B_{1}^{+}$ on the other (Figure 3A). This asymmetry in the transverse plane is a known effect for surface coils in UHF MRI and has been well explained by Vaidya et al. [56]. In fact, the induced conduction currents which are stronger at higher frequencies create magnetic field contributions that are 90° out of phase with the one directly generated by coil elements. When these contributions interact orthogonally with the primary magnetic field they can contribute more to either left or right circular polarisation based on their geometrical disposition.

Single‐loop coils are known to exhibit this behaviour. However, a better understanding requires considering the current distribution in the whole array. As the *X*‐direction currents at the intersection of loop elements compensate and cancel each other, the array behaves as two overlapped loops with 180° phase difference, or to simplify even more, as a set of 4 active dipoles. Currents on the interior dipoles (representing the central conductive segments where loop overlaps) would then flow in the opposite direction from currents on the exterior dipoles (representing the outer edges of the array). The simulated 4‐dipole model validates this analogy as it exhibits a $B_{1}^{+}$ pattern matching the one created by the loop array Figure 3B. The asymmetry described before is still present (although it has been shown that $B_{1}^{+}$ patterns of a single dipole antenna exhibit no twisting [57], this is not necessarily the case for an array of dipoles, especially when excitation modes create current loops).

This 4‐dipole model provides a better understanding of the role of the two 600 mm passive dipoles presented in this study. As shown by Figure 4B, their dimension greater than their resonance length makes them respond in phase opposition to the currents of the exterior dipoles (and so in phase with the currents of the interior dipoles). Thus, they can be considered to act as ‘reflectors’ in analogy with those used in telecommunication antennas [58]. Their contributions interfere with those of the active elements and cancel the spurious magnetic field lobes. As illustrated in Figure 4A, for the most favourable 600 mm case the current circulating in the middle of the passive dipoles has the same amplitude as the current circulating in the outer edge of the loops: both contributions compensate each other. As a result, $B_{1}^{+}$ was focused in the central region (Figure 3C,D), which led to gains in homogeneity and transmit efficiency in the ROI. Limiting the unwanted illumination outside of the ROI could also help to attenuate fold‐over artifacts that occur when using small field‐of‐view (FoV) or even advanced reduced FoV imaging [59].

The ‘phase opposition’ passive elements used in this study are very case‐specific as they must adapt to the active array shape and ROI geometry. The proposed design is thus conceptually different from most passive structures previously investigated in MRI where the main goal was to obtain an in‐phase response with the main coil to contribute constructively to the transmit field [60]. The passive elements used in this work rely on principles similar to the previously proposed use of destructive interference of dipole arrays for better field control [61], or the usage of directors and reflectors in analogy with Yagi‐Uda arrays [62].

Although adding dipoles lowers total accepted power (Table 1) by acting as parasitic bridges that increase cross‐coupling and load dissipation, transmit efficiency significantly improves. While the baseline array accepts more power, much is wasted on undesired lateral field spread. The passive dipoles suppress this lateral radiation, spatially concentrating the remaining radiated power into the target region for a superior efficiency ratio. Additionally, the SAR reduction predicted by the simulations (Table 1) is consistent with the analysis developed by Zivkovic et al. [38] which explains that passive dipoles can lower SAR by shielding the sample from the high conservative electric fields of the active elements. They also state that segmented passive dipoles have reduced inductive field thanks to a more homogeneous current distribution, which explains the interest of adding a lumped capacitor. Solomakha et al. [36] also observed that the even modes of dipole arrays (where all currents shared the same direction) exhibited lower SAR than the odd modes (with currents flowing in opposite directions): in our case, adding the passive dipoles reinforces an even mode. Importantly, as detailed in the Supporting Information S3, these significant transmit advantages are achieved without compromising the receive performance or parallel imaging stability of the underlying loop array.

The proposed design addresses a broader architectural trade‐off in 7T body coil development. While two‐channel active dipole antennas can significantly outperform standard eight‐channel loop arrays in $B_{1}^{+}$ and SAR efficiency [20], our supplementary analysis (Supporting Information S4) demonstrates that the integration of passive dipoles effectively narrows this performance gap. By achieving dipole‐level efficiency while preserving an 8‐channel transmit density, this architecture combines the deep penetration of dipole antennas with the high degrees of freedom that are necessary for subject‐specific shimming and parallel transmit (pTx).

Regarding passive dipole segmentation, the 10 pF capacitors used in this prototype were based on preliminary evaluations. A subsequent finer analysis (Supporting Information S5) revealed a performance trade‐off: higher capacitances (e.g., 50 pF) maximised $B_{1}^{+}$ efficiency, while lower capacitances (e.g., 5 pF) maximised SAR efficiency. While the 10‐pF configuration serves as an effective proof‐of‐concept for the macroscopic field‐shaping mechanism, future iterations can be fine‐tuned with lower lumped capacitances to further improve experimental performance.

Selecting dipoles longer than dimensions of the loop array was shown to be desirable for the homogenisation of $B_{1}^{+}$ field as well as the extension of transmit performance in the longitudinal direction. While the 30 cm phantom ROI was selected to balance cord coverage with B0/gradient linearity and acquisition times, simulations indicated further improved transmit performance for a 40 cm ROI. This extended longitudinal coverage could enable multi‐FoV imaging of the entire thoraco‐lumbar spine without patient repositioning. As the passive dipoles can be integrated in the table bed, there may be less constraints on their dimensions than for head or cervical designs.

Additionally, the mechanical integration of the passive dipoles presents a distinct structural advantage. In a clinical thoracic spine array, high‐power active transmit loops must be housed beneath a rigid, load‐bearing cover plate for weight support and thermal/SAR safety, inherently creating a sizable gap from the patient. However, because the passive dipole elements can be fabricated on thin, flexible substrates, they are not constrained by the rigid housing and can be embedded much closer to the subject, directly beneath the surface padding. As confirmed by our supplementary air‐gap analysis, this closer proximity can be safely achieved without generating adverse local SAR hotspots. This robustness is vital when extrapolating these static phantom results to in vivo imaging. Real clinical scenarios introduce inter‐patient anatomical variability—such as differences in BMI or spinal curvature—which will alter the effective distance and coupling between the coil and the target anatomy. The coil's stable performance across various airgaps suggests that the fundamental mechanism of passive field reshaping will translate effectively despite these variations.

Further studies are required to evaluate this design in vivo, and to measure the electric field to characterise SAR levels and further validate safety in the presence of human tissue heterogeneity. Because anatomical variations will inevitably induce patient‐specific $B_{1}^{+}$ phase and amplitude alterations, combining the proposed hardware‐based $B_{1}^{+}$ redistribution method with active software methods such as pTx would be of great interest. Future work will determine if this hybrid architecture can offer improved pTx capabilities and further SAR reduction, or if its ability to pre‐focus the field on the spinal cord region could enable faster computation time for tailored RF pulses.

## 5 Conclusion

The main objective of this study was to optimise the $B_{1}^{+}$ transmit field distribution for thoracic spinal cord imaging at 7T. To achieve this, a novel coil design was proposed, combining an 8‐channel loop array with two passive, out‐of‐phase dipoles positioned under its outer edges. By reshaping the $B_{1}^{+}$ field to suppress spurious lateral contributions and increase penetration depth, this combined configuration significantly improved key transmit metrics compared to the loop array alone. Accordingly, experimental proof‐of‐concept results obtained with a homogeneous phantom showed a 25% gain in $B_{1}^{+}$ efficiency and a 16% gain in $B_{1}^{+}$ homogeneity within the ROI. Furthermore, numerical simulations using a human voxel model predicted a 35% gain in SAR efficiency in the voxel‐model ROI.

## Funding

This work received support from the French Government under the France 2030 Investment Plan and French ‘Investissements d'Avenir’ programme, as part of the Initiative d'Excellence d'Aix‐Marseille Université, AMIDEX: AMX‐19‐IET‐002, AMX‐23‐CI‐01, AMX‐23‐CPJ‐10 and AMX‐23‐EQ‐FO‐009 as well as from the Agence Nationale de la Recherche: ANR‐23‐CE17‐0019‐01 and from CNRS Ingeniere: PEPS2024.

## Conflicts of Interest

Hugo Amat, Djamel Berrahou and Amira Trabelsi are currently employed by Multiwave Technologies SAS. Hugo Amat is supported through a French CIFRE industrial PhD fellowship. While primarily focused on developing MRI hardware for ultra‐low‐field applications, the company has also conducted research and holds intellectual property in the domain of high‐ and ultra‐high‐field. The remaining authors declare no conflicts of interest.

## Supporting information


**Table S1:** Numerical evaluation of the distance between passive dipoles, simulations with homogenous phantom.
**Table S2:** Numerical evaluation of the airgap between the skin and the passive dipoles, simulations with Gustav voxel‐model.
**Figure S3.1:** Noise correlation matrices for the array only (left) and the array with passive dipoles (right).
**Figure S3.2:** Raw SNR (top) and $B_{1}^{+}$‐corrected SNR (bottom). The enhancement ratio map is obtained through a pixel‐by‐pixel division of the ‘Array + Dipoles’ map by the ‘Array Only’ map.
**Figure S3.3:** Inverse g‐factor maps, axial acquisition, 1D acceleration along A‐P direction.
**Figure S3.4:** Inverse g‐factor maps, sagittal acquisition, 1D acceleration along H‐F direction. Note: The localised variations and gradients in 1/g observed in the [0, 150] longitudinal region at higher acceleration factors are recognised artifacts of the SENSE unfolding algorithm, driven by the prescribed FOV (600 mm) exceeding the physical length of the phantom (480 mm). At specific aliasing distances, the unfolding matrix is forced to separate tissue signal from zero‐signal air regions outside the phantom, creating artificial local gradients that do not reflect the intrinsic parallel imaging capability of the array.
**Table S4:** Performance comparison between the loop array (with and without passive dipoles) and a double Tx dipole antenna, simulations with homogeneous phantom.
**Table S5:** Numerical evaluation of the segmentation capacitance of passive dipoles, simulations with homogenous phantom.

## Data Availability

The data that support the findings of this study are available from the corresponding author upon reasonable request.
