# Supplementary material for: Enhanced Transmit Efficiency of a Loop Array Combined With Out‐of‐Phase Passive Dipoles for Thoracic Spinal Cord MRI at 7T
Source: Magn Reson Med. 2026 Jul 26;96(5):2539–52. doi: 10.1002/mrm.70517 (PMC13527284; doi:10.1002/mrm.70517)
Supplement: Supplementary file 1 [file MRM-96-2539-s001.docx]

Supporting Information

# S1 – Effect of distance between passive dipoles

In the design featured , the passive dipoles are separated by 150 mm along the X or left-right axis. This dimension was geometrically selected to align the out-of-phase passive elements directly beneath the outer edges of the active loop array to maximize lateral field suppression. To evaluate the effect of lateral spacing on coil performance, a numerical analysis was conducted.

Full-wave electromagnetic simulations were performed comparing the reference 150 mm spacing against a narrower (130 mm) and wider (170 mm) configuration (Table S3). Bringing the passive dipoles closer together (130 mm) increases raw $B_{1}^{+}$efficiency but simultaneously causes a sharp increase in the maximum local 10g SAR. Conversely, the 150 mm spacing provides a balanced operating point, mitigating peak SAR while maintaining good field homogeneity. Consequently, the 150 mm spacing coincides with the peak SAR efficiency of the tested configurations.

Table S1: Numerical evaluation of the distance between passive dipoles, simulations with homogenous phantom

|  | Array only  (reference) | Array & 600 mm passive dipoles  130 mm spacing between dipoles | Array & 600 mm passive dipoles  150 mm spacing between dipoles | Array & 600 mm passive dipoles  170 mm spacing between dipoles |
| --- | --- | --- | --- | --- |
| Pstim (W) | 1 | 1 | 1 | 1 |
| Pacc (W) | 0,99 | 0.92 | 0,85 | 0.85 |
| B_1_^+^ Avg ($\mu T/\sqrt{W}$) in ROI | 0,209 | 0.256 | 0,229 | 0.218 |
| CoV in ROI | 0,40 | 0,32 | 0,31 | 0.32 |
| Max SAR 10g (W/kg) for the whole phantom | 1,29 | 1.28 | 0,98 | 0.97 |
| $\frac{\text{B}\text{1}\text{+}\text{ avg (ROI) }}{\sqrt{\text{max SAR (phantom) }}}$ ($\mu T/\sqrt{W/kg})$ | 0,184 | 0.226 | 0,231 | 0.221 |

# S2 – Robustness to airgap variations between the patient and the antenna

To evaluate the robustness of the design against variations in patient positioning, clothing, or padding thickness, different airgaps were evaluated. Using the Gustav voxel model, the entire coil ensemble was shifted relative to the subject to simulate variable padding thicknesses. This resulted in effective dipole-to-skin airgaps of 2 mm (minimal padding), 5 mm (the reference configuration), 10 mm (thicker padding), and 15 mm (an extreme case where the passive dipoles are pushed as far back as the active loop array is in the reference case).

As shown in Table S4, the performance of the hybrid array is robust across the entire 2 mm to 15 mm range. $B_{1}^{+}$ efficiency, SAR efficiency, and field homogeneity remain stable regardless of proximity. Notably, reducing the dipole airgap to 2 mm does not induce a significant spike in the peak local 10g SAR. This stability occurs because the distance is measured from the extremities of the shoulder blades to the passive dipoles, whereas the actual SAR hotspot remains centrally located between the shoulder blades, directly beneath the active loop array. This demonstrates that placing the passive dipoles in close proximity to the subject within the padding layer is both safe and electromagnetically stable.

Table S2: Numerical evaluation of the airgap between the skin and the passive dipoles, simulations with Gustav voxel-model

|  | Array only  (reference) | Array & passive dipoles  Airgap = 2 mm | Array & passive dipoles  Airgap = 5 mm | Array & passive dipoles  Airgap = 10 mm | Array & passive dipoles  Airgap = 15 mm |
| --- | --- | --- | --- | --- | --- |
| Pstim (W) | 1 | 1 | 1 | 1 | 1 |
| Pacc (W) | 0,93 | 0,69 | 0,71 | 0,75 | 0,78 |
| B_1_^+^ Avg ($\mu T/\sqrt{W}$) in ROI | 0,194 | 0,216 | 0,216 | 0,214 | 0,212 |
| CoV in ROI | 0,56 | 0,38 | 0,38 | 0,37 | 0,37 |
| Max SAR 10g (W/kg) for the whole phantom | 1,01 | 0,69 | 0,69 | 0,67 | 0,65 |
| $\frac{\text{B}\text{1}\text{+}\text{ avg (ROI) }}{\sqrt{\text{max SAR (phantom) }}}$ ($\mu T/\sqrt{W/kg})$ | 0,193 | 0,260 | 0,260 | 0,261 | 0,263 |

# S3 – Rx performance

The antenna was tested in a 7T scanner (Magnetom TERRA, Siemens Healthcare, Erlangen, Germany) using a rectangular phantom filled with body mimicking solution (Body Liquid 300, MVG) of dimensions 350 × 175 × 450 mm³ and dielectric properties εr = 58.2, σ = 0.92 S·m⁻¹. An eight-channel T/R switch box (Stark Contrast, Erlangen, Germany) was used as an interface connection between the array and the scanner. Two different configurations were tested: one with the loop array only placed on the phantom, and one with passive dipoles (600 mm long, 10 pF segmentation capacitance) placed between the loop array and the phantom.

To test the receive performance and parallel imaging capabilities of the array, the vendor-provided coil utility product sequence available on the Siemens Terra 7T platform was utilized (software version VE12U). This tool, which is based on the foundational coilQA framework originally developed by Massachusetts General Hospital, employs a 2D gradient echo sequence to simultaneously acquire data for the evaluation of the noise covariance, SNR, and simulated SENSE inverse g-factors^1^. Specifically, noise covariance matrices were extracted to verify that the passive dipoles do not introduce detrimental inductive coupling between the individual receive loops, while inverse g-factors were evaluated to quantify the stability of the spatial encoding and ensure parallel imaging capabilities remain uncompromised. To ensure quantitative accuracy, the sequence reconstructs optimal SNR maps using a noise-covariance-weighted root-sum-of-squares combination^2^ and applies a spatial correction to account for Rician magnitude bias^3^. For the axial orientation, data were acquired with 1.0 × 1.0 × 2.5 mm³ resolution, 384 × 384 mm² field of view (FOV), 15 slices, TR/TE = 30/6 ms, nominal flip angle = 12°, phase encoding in the Antero-Posterior (A-P) direction, and a total acquisition time of 2 min 52 s. For the sagittal orientation, parameters were adjusted to accommodate the longitudinal extent of the spine: 3.1 × 3.1 × 2.0 mm³ acquired resolution, 600 × 600 mm² FOV, 13 slices (with a 20% slice gap), TR/TE = 30/6 ms, flip angle = 12°, phase encoding in the Head-Foot (H-F) direction, and a total acquisition time of 1 min 15 s.

The SNR maps were evaluated using a sagittal acquisition rather than an axial one, as the chosen sagittal spatial resolution and geometric alignment allowed for increased signal penetration and maximized baseline SNR at depth. To ensure quantitative accuracy, the reported mean SNR values were computed volumetrically across the full 3D ROI. To be able to compute $B_{1}^{+}$-compensated SNR maps, 3D Actual Flip Angle Imaging (AFI) was also utilized. The sequence was acquired with a coronal orientation using the following parameters: spatial resolution = 5 × 5 × 5 mm³, FOV = 200 × 240 mm², block repetition time (TR) = 146 ms, nominal flip angle = 60°, and total acquisition time = 4 min 30. The AFI reconstruction included $B_{0}$ correction to ensure robust flip angle mapping despite magnetic field inhomogeneities at the edges of the phantom in the Z direction. The raw SNR data was then pixel-wise divided by the sine of the local, measured flip angle. This compensation mathematically removes the $B_{1}^{+}$ transmit bias, giving maps that accurately reflect the true receive performance of the array both with and without the passive dipoles. To prevent mathematical divergence during pixel-wise division, the $B_{1}^{+}$-compensated SNR and enhancement ratio maps were masked to exclude regions with inadequate flip angle. Consequently, the longitudinal extent of the quantitative 3D ROI was adjusted to 200 mm to ensure only robustly encoded voxels were evaluated. Reported mean $B_{1}^{+}$-compensated SNR values were then computed volumetrically across this 3D ROI.

The parallel imaging capabilities of the array were quantified using the inverse g-factor. For each axial or sagittal slice, the mean inverse g-factor and the 5^th^ percentile were computed in the corresponding 2D section of the ROI (30 × 30 × 300 mm^3^, located on a central position in the XZ plane and 50 mm deep under the surface of the phantom).

Figure S3.1: Noise correlation matrices for the array only (left) and the array with passive dipoles (right)

**Noise Covariance:** The addition of the passive dipoles resulted in a minor, expected increase in inter-element noise correlation (average correlation rising from 8.5% to 14.3%). This level of inter-element isolation remains within acceptable limits for a 8-channel 7T array.

Figure S3.2: Raw SNR (top) and $B_{1}^{+}$-corrected SNR (bottom). The enhancement ratio map is obtained through a pixel-by-pixel division of the “Array + Dipoles” map by the “Array Only” map.

**Signal-to-Noise Ratio (SNR):** The raw SNR maps (defined here as the uncorrected SNR extracted directly from the reconstructed images, which inherently encapsulates the combined effects of both the achieved local flip angle and the intrinsic receive sensitivity) exhibited a net average improvement of +9% within the 3D target ROI, driven by the transmit efficiency gains. Transmit-compensated SNR maps confirms that the intrinsic receive sensitivity experienced only a marginal reduction (–4.6%), demonstrating that the array's transmit efficiency gains translate effectively to a net-positive final image SNR.

Figure S3.3: inverse g-factor maps, axial acquisition, 1D acceleration along A-P direction.

Figure S3.4: inverse g-factor maps, sagittal acquisition, 1D acceleration along H-F direction.

**Parallel Imaging (-factors):** Inverse SENSE g-factor maps demonstrate that the spatial encoding integrity of the 4x2 array is preserved upon dipole integration. The array maintained robust unfolding capabilities up to in the axial plane (A-P acceleration) and in the sagittal plane (H-F acceleration), with negligible degradation in the 5th percentile limits inside the spinal cord ROI.

# S4 – Benchmark Comparison: Loop array with passive dipoles vs. Double Active Dipole Array

To evaluate the field distribution and efficiency of the proposed design against an alternative deep-penetration architecture, a comparative simulation was performed using a benchmark double active dipole array (adapted from Duan et al., MRM 2015^4^). The two active dipoles (length = 447 mm, corresponding to their resonant length in this loaded environment) were positioned 2 cm above the homogeneous phantom with an optimized lateral spacing of 75 mm to balance constructive interference and mutual coupling (S21 = -12.5 dB).

It should be noted that this benchmark represents a baseline dual-dipole model used strictly for comparative evaluation of field distributions, and does not incorporate advanced dual-mode or fractionated geometries. Note also that to ensure a fair comparison of maximum theoretical capabilities (and various phase offsets significantly , all designs in this benchmark evaluation (including our 8-loop array) were evaluated at their optimized lateral peak field positions. This accounts for the higher baseline efficiencies reported in this table compared to the geometrically-centered configurations in the main manuscript.

Table S4: Performance comparison between the loop array (with and without passive dipoles) and a double Tx dipole antenna, simulations with homogeneous phantom

|  | 8-loop array | 8-loop array & 600 mm passive dipoles | Double Tx dipole  270° phase offset | Double Tx dipole  300° phase offset | Double Tx dipole  330° phase offset | Double Tx dipole  0° phase offset | Double Tx dipole  30° phase offset |
| --- | --- | --- | --- | --- | --- | --- | --- |
| Pstim (W) | 1 | 1 | 1 | 1 | 1 | 1 | 1 |
| Pacc (W) | 0.99 | 0,85 | 0,94 | 0,94 | 0,93 | 0,93 | 0,93 |
| B_1_^+^ Avg ($\mu T/\sqrt{W}$) in ROI | 0.225 | 0,249 | 0,259 | 0,250 | 0,235 | 0,227 | 0,227 |
| CoV | 0.38 | 0,30 | 0,32 | 0,31 | 0,31 | 0,31 | 0,32 |
| Max SAR 10g (W/kg) for the whole phantom | 1.30 | 0,98 | 1,28 | 1,14 | 0,95 | 0,75 | 0,95 |
| $\frac{\text{B}\text{1}\text{+}\text{ avg (ROI) }}{\sqrt{\text{max SAR (phantom) }}}$ ($\mu T/\sqrt{W/kg})$ | 0.197 | 0,252 | 0,228 | 0,234 | 0,241 | 0,262 | 0,233 |

A phase sweep demonstrated that exciting the double active dipoles in-phase (0° offset) yielded the highest SAR efficiency (0.262 $\mu T/\sqrt{W/kg}$). While this benchmark configuration demonstrates significantly higher raw efficiency (33% better) than the baseline 8-channel loop array (0.197 $\mu T/\sqrt{W/kg}$), the addition of the passive dipoles to the loop array elevates its SAR efficiency (to 0.252 $\mu T/\sqrt{W/kg}$), effectively closing the performance gap (a difference of 4%). Moreover, the loop array with passive dipoles offers higher $B_{1}^{+}$ efficiency (0.249 $\mu T/\sqrt{W}$) than the double active dipoles with 0° phase offset (0.227 $\mu T/\sqrt{W}$). Consequently, the hybrid design achieves dipole-level deep penetration efficiency while retaining the dense transmit channel count required for pTx applications.

# S5 –Effect of passive dipole segmentation capacitance

To characterize the influence of the passive dipoles lumped segmentation capacitance on the transmit performance, a parametric analysis was conducted.

Numerical simulations were conducted for the loop array with 600 mm passive dipoles placed above the homogeneous phantom. As shown in Table S2.1, adjusting the segmentation capacitance alters the balance between $B_{1}^{+}$ efficiency and SAR efficiency. While higher capacitance values (e.g., 50 pF) maximize raw $B_{1}^{+}$ efficiency, lower values (e.g., 2 pF to 5 pF) minimize SAR and maximize SAR efficiency. The 10 pF configuration acts as a robust intermediate operating point, delivering near-optimal SAR efficiency alongside highly competitive $B_{1}^{+}$ efficiency and field homogeneity.

Table S5: Numerical evaluation of the segmentation capacitance of passive dipoles, simulations with homogenous phantom

|  | Array only  (reference) | Array & 600 mm passive dipoles  1 pF | Array & 600 mm passive dipoles  2 pF | Array & 600 mm passive dipoles  5 pF | Array & 600 mm passive dipoles  10 pF | Array & 600 mm passive dipoles  20 pF | Array & 600 mm passive dipoles  50 pF | Array & 600 mm passive dipole  shortcut |
| --- | --- | --- | --- | --- | --- | --- | --- | --- |
| Pstim (W) | 1 | 1 | 1 | 1 | 1 | 1 | 1 | 1 |
| Pacc (W) | 0,99 | 0,93 | 0,87 | 0,84 | 0,85 | 0,85 | 0,86 | 0,86 |
| B_1_^+^ Avg ($\mu T/\sqrt{W}$) in ROI | 0,209 | 0,183 | 0,190 | 0,217 | 0,229 | 0,232 | 0,235 | 0,234 |
| CoV in ROI | 0,40 | 0,39 | 0,34 | 0,30 | 0,31 | 0,32 | 0,33 | 0,33 |
| Max SAR 10g (W/kg) for the whole phantom | 1,29 | 0,87 | 0,73 | 0,83 | 0,98 | 1,05 | 1,12 | 1,14 |
| $\frac{\text{B}\text{1}\text{+}\text{ avg (ROI) }}{\sqrt{\text{max SAR (phantom) }}}$ ($\mu T/\sqrt{W/kg})$ | 0,184 | 0,196 | 0,222 | 0,238 | 0,231 | 0,225 | 0,222 | 0,219 |

# References

1.	Pruessmann KP, Weiger M, Scheidegger MB, Boesiger P. SENSE: Sensitivity encoding for fast MRI. *Magnetic Resonance in Medicine*. 1999;42(5):952-962. doi:10.1002/(SICI)1522-2594(199911)42:5<952::AID-MRM16>3.0.CO;2-S

2.	Roemer PB, Edelstein WA, Hayes CE, Souza SP, Mueller OM. The NMR phased array. *Magnetic Resonance in Medicine*. 1990;16(2):192-225. doi:10.1002/mrm.1910160203

3.	Kellman P, McVeigh ER. Image reconstruction in SNR units: A general method for SNR measurement. *Magnetic Resonance in Medicine*. 2005;54(6):1439-1447. doi:10.1002/mrm.20713

4.	Duan Q, Nair G, Gudino N, et al. A 7T spine array based on electric dipole transmitters. *Magn Reson Med*. 2015;74(4):1189-1197. doi:10.1002/mrm.25817
